# Early Clinical Response after 2 Weeks of Sorafenib Therapy Predicts Outcomes and Anti-Tumor Response in Patients with Advanced Hepatocellular Carcinoma

**DOI:** 10.1371/journal.pone.0138776

**Published:** 2015-09-30

**Authors:** Teiji Kuzuya, Masatoshi Ishigami, Yoji Ishizu, Takashi Honda, Kazuhiko Hayashi, Yoshiaki Katano, Yoshiki Hirooka, Tetsuya Ishikawa, Isao Nakano, Hidemi Goto

**Affiliations:** 1 Department of Gastroenterology and Hepatology, Nagoya University Graduate School of Medicine, Nagoya, Japan; 2 Department of Gastroenterology, Banbuntane Hotokukai Hospital, Fujita Health University, School of Medicine, Nagoya, Japan; University of Navarra School of Medicine and Center for Applied Medical Research (CIMA), SPAIN

## Abstract

**Background & Aims:**

We evaluated the relationship between the early clinical response after 2 weeks of sorafenib therapy and the outcomes and anti-tumor response in patients with advanced hepatocellular carcinoma.

**Methods:**

Fifty-seven patients who had intrahepatic hypervascular hepatocellular carcinoma and Child-Pugh (CP) class A disease at baseline were enrolled in this prospective, multicenter, observational, non-interventional study. As an early clinical response after 2 weeks of sorafenib therapy, changes in intra-tumor blood flow on contrast-enhanced computed tomography (CE-CT), alpha-fetoprotein (AFP) levels, and remnant liver function were investigated.

**Results:**

After 2 weeks of sorafenib therapy, there were 26 patients (45.6%) without disappearance of arterial tumor enhancement on CE-CT, 15 patients (26.3%) with an AFP ratio of >1.2, and seven patients (12.3%) with two or more increments in the CP score. Multivariate analysis showed that the absence of disappearance of arterial tumor enhancement on CE-CT, AFP ratio of >1.2, and two or more increments in the CP score after 2 weeks of sorafenib therapy were significant and independent predictors of worse survival. Upon scoring these three variables as "poor prognostic factors", patients with poor prognostic score 4, 3 or 2 (n = 17) had significantly worse outcomes and a significantly higher progressive disease (PD) rate based on modified Response Evaluation Criteria in Solid Tumors at 6 weeks after sorafenib therapy than those with poor prognostic score 1 or 0 (n = 40) (median overall survival: 194 days vs. 378 days; p = 0.0010, PD rate: 70.6% vs. 20.0%; p = 0.0003, respectively).

**Conclusions:**

Changes in intra-tumor blood flow on CE-CT, AFP levels, and remnant liver function after 2 weeks of sorafenib therapy may be useful for predicting the outcomes and anti-tumor response to sorafenib in patients with advanced hepatocellular carcinoma.

## Introduction

Sorafenib is a molecularly targeted multi-kinase inhibitor that suppresses the signal transduction pathways that mediate tumor growth and angiogenesis. Large-scale phase III clinical studies demonstrated that sorafenib significantly improved overall survival (OS) and time-to-progression in patients with advanced hepatocellular carcinoma (HCC) [1,2]. Accordingly, sorafenib has been widely used as the only first-line standard systemic agent currently available for the treatment of advanced unresectable HCC [3–7].

However, sorafenib therapy has several limitations. First, sorafenib therapy is associated with a low objective response rate and limited prolongation of survival [1,2]. Second, sorafenib therapy is associated with various kinds of drug-related adverse events that affect almost all treated patients [1,2,4,8]. Finally, sorafenib therapy is very expensive [9]. Therefore, there is an urgent medical need for the early identification of patients who are expected to benefit from sorafenib therapy. This effort could also help identify patients who are not good candidates for the drug, and in whom withholding the drug could therefore prevent needless adverse events and costs.

Several studies have investigated prognostic factors and serum biomarkers to predict treatment outcomes for patients treated with sorafenib [10–14]. Disappearance of tumor staining on contrast-enhanced computed tomography (CE-CT) [15] or on contrast-enhanced ultrasound [16,17], decrease in alpha-fetoprotein (AFP) level [18–21] and decrease in vascular endothelial growth factor level [22] have been reported as indicators of good prognosis and the anti-tumor effect in the early post-dose period of sorafenib therapy.

Our early experience of sorafenib therapy also showed a good anti-tumor effect and prognosis in patients whose tumor staining had disappeared on CE-CT and whose AFP level had decreased in the early post-dose period (i.e., the first 2 weeks post-dose) [23,24]. Conversely, we also observed patients with deterioration in hepatic reserve and poor prognosis in the early post-dose period. However, there have been no established reports of clinically useful predictive biomarkers in the very early period following administration of sorafenib to patients with advanced HCC.

Based on our early post-dose results, we conducted a prospective study to investigate the hypothesis that clinical changes (i.e., changes in tumor staining, AFP, and hepatic reserve) in the early post-dose period (i.e., 2 weeks post-dose) of sorafenib therapy are useful indicators of prognosis and therapeutic response.

## Patients and Methods

### Study Design

This was a prospective, multicenter, observational, non-interventional study to investigate the early clinical response after 2 weeks of sorafenib (Nexavar, Bayer Yakuhin, Ltd., Osaka, Japan) therapy and outcomes in patients with advanced HCC. The primary endpoint was the relationship between early changes and overall survival, and the secondary endpoint was the relationship between early changes and the anti-tumor effect. This study was approved by the ethics committees of each participating hospital; in Nagoya University School of Medicine, Fujita Health University, Ogaki Municipal Hospital, Okazaki City Hospital, Kariya Toyota General Hospital, Tosei General Hospital, Komaki City Hospital, Anjo Kosei Hospital, Kainan Hospital, Toyota Kosei Hospital, Yokkaichi Municipal Hospital, Nagoya Medical Center, Toyohashi Municipal Hospital, Japanese Red Cross Nagoya Daiichi Hospital, Handa City Hospital, Ichinomiya Municipal Hospital, Konan Kosei Hospital, and Toyota Memorial Hospital. Written informed consent was obtained from each patient, and this study was performed in compliance with the 1975 Declaration of Helsinki.

### Study Patients

Patients who satisfied all of the inclusion criteria and who did not meet any of the exclusion criteria were eligible to enroll in this study.

The inclusion criteria were as follows: 1) typical HCCs diagnosed clinically using CE-CT imaging based on guidelines established by the American Association for the Study of Liver Diseases [25]; 2) at least one measurable and hypervascular HCC with diameter of over 10 mm in the liver at baseline; 3) patients who were not eligible for surgical resection or locoregional therapy; 4) Child-Pugh (CP) class A disease in terms of remnant liver function; 5) Eastern Cooperative Oncology Group Performance Status (ECOG PS) of 0 or 1; 6) neutrophil count >1500 /μL, platelet count >50,000 /μL, hemoglobin ≥8.5 g/dL, total bilirubin <2.0 mg/dL, serum aspartate aminotransferase and serum alanine aminotransferase <5 times the upper limit of the study center standard range, serum creatinine ≤1.5 times the upper limit of the study center standard range; 7) serum AFP ≥20 ng/ml; and 8) age of at least 20 years at the time of enrollment.

Exclusion criteria were as follows: 1) scheduled to undergo concomitant therapy with another anti-cancer agent; 2) history of systemic chemotherapy; 3) active multiple cancers; 4) sorafenib treatment not recommended for various reasons (e.g., brain tumor present, on dialysis, women who are or may be pregnant, poorly controlled hypertension, myocardial infarction, unstable angina, cardiac failure, esophageal varices with the potential to bleed, or cerebrovascular disorder in the 12-month period before enrollment); and 5) CE-CT not feasible due to a contraindication against radiographic contrast media or another reason.

Between August 2011 and July 2013, a total of 123 patients (among 18 institutions) receiving sorafenib therapy were screened for this study. Of the 123 patients, the following were excluded from this study: patients with CP class B disease (n = 8); patients with laboratory values not within the data range (n = 11); patients with serum AFP <20 ng/ml (n = 33); patients without hypervascular HCC in the liver (n = 6); patients in whom CE-CT could not be performed for any reason (n = 7); and patients receiving dialysis therapy (n = 1). The remaining 57 patients were enrolled in this study. Follow-up analysis was terminated at the end of July 2014.

### Sorafenib Therapy

The starting dose of sorafenib was 800 mg/day administered orally. However, out of concern for the possibility of having to discontinue sorafenib treatment at an early stage due to adverse events, the initial dose was set at 400 mg/day for patients who were 80 years or older, for those who had a body weight of 40 kg or less, for those with poor renal function, and for those who had a history of treatment for varices or ascites. Adverse events were assessed according to the Common Terminology Criteria for Adverse Events version 4.0. In the case of the occurrence of drug-related adverse events, a dose reduction or temporary interruption was maintained until the symptoms resolved to grade 1 or 2 according to the guidelines provided by the manufacturer. Sorafenib therapy was continued until the occurrence of potentially fatal adverse events or clinical tumor progression.

### Evaluation of Anti-Tumor Response

Four-phase CE-CT scans (i.e., unenhanced, late arterial, portal venous, and equilibrium phase) were obtained at baseline, at 2 and 6 weeks after sorafenib administration, and then every 4–8 weeks. Anti-tumor responses were evaluated according to the modified Response Evaluation Criteria in Solid Tumors (RECIST) [26]. Before sorafenib administration, measurable and hypervascular HCCs with diameters of over 10 mm at baseline were defined as the target lesions. When more than five lesions were present, a maximum of five lesions were defined as target lesions. Anti-tumor responses of all enrolled patients were blindly assessed by two independent evaluators. In cases of discrepancies, the CE-CT images were evaluated jointly by both evaluators, and a consensus decision was reached. Initial evaluations of anti-tumor responses were performed at 6 weeks after sorafenib administration.

### Evaluation of Changes in Intra-Tumor Blood Flow at 2 Weeks after Sorafenib Administration

In order to evaluate changes in intra-tumor blood, we investigated whether or not arterial tumor enhancement on CE-CT disappeared at 2 weeks after sorafenib administration. The absence of disappearance of intra-tumor blood flow was defined as tumor staining in the target lesion that showed absolutely no sign of disappearing on CE-CT at 2 weeks post-dose when compared with staining on CE-CT at baseline. Changes in intra-tumor blood flow of all enrolled patients were also blindly assessed by two independent evaluators. In cases of discrepancies, the CE-CT images were evaluated jointly by both evaluators, and a consensus decision was reached. In the present study, assessment of images at 6 weeks post-dose were used to evaluate the initial anti-tumor effect so that assessment at 2 weeks post-dose did not result in any changes to the sorafenib dose.

### Evaluation of Changes in AFP Level at 2 Weeks after Sorafenib Administration

The analyzed HCC tumor marker was serum AFP at baseline and at 2 weeks after starting sorafenib administration. For each patient, the baseline AFP level was assigned a value of 1, and the ratios for AFP level at 2 weeks relative to baseline AFP level after the start of administration were calculated. A high AFP ratio was defined as an AFP ratio of >1.2 at 2 weeks after sorafenib administration [27,28].

### Evaluation of Changes in Remnant Liver Function at 2 Weeks after Sorafenib Administration

We evaluated the changes in CP score as changes in remnant liver function. Deterioration of liver function was defined as two or more increments in the CP score at 2 weeks after sorafenib administration.

### Statistical Analysis

Statistical analyses were performed using Stat View J Ver. 5 software (SAS Institute, Cary, NC, USA). Overall survival (OS) was measured from the start date of sorafenib therapy until the date of death or date of last visit. OS after the start of sorafenib administration was calculated by the Kaplan-Meier method, and differences in survival were evaluated by the log-rank test. The Cox proportional hazard model was used to identify prognostic factors. Factors with a *p* value < 0.05 at univariate analysis were included in the final multivariate model. Categorical variables were analyzed using a Fisher’s exact probability test. For all analyses, *p* value of <0.05 was considered to indicate a statistically significant difference.

## Results

### Baseline Patient Characteristics

Table 1 summarizes the baseline characteristics of the 57 HCC patients enrolled in this study. Patients consisted of 46 males and 11 females, with a median age of 71 years (range, 35–83 years). There were 35 patients with a CP score of 5 and 22 patients with a CP score of 6. The median baseline AFP level was 491 ng/ml (range, 21–315,700 ng/ml).

**Table 1:** Baseline characteristics of 57 patients with hepatocellular carcinoma.

Characteristics	n = 57
Median age (years, range)	71 (35–83)
Gender (male/female), n (%)	46 (80.7) / 11 (19.3)
Etiology (HBV/HCV/NBNC), n (%)	8 (14.0) /29 (50.9) / 20 (35.1)
ECOG PS (0/1), n (%)	41 (71.9) / 16 (28.1)
Initial therapy/therapy for recurrence, n (%)	9 (15.8) / 48 (84.2)
Child-Pugh score (5/6), n (%)	35 (61.4) / 22 (38.6)
Tumor size (<30 mm/≥30 mm), n (%)	22 (38.6) / 35 (61.4)
Number of tumors (<4/≥4), n (%)	10 (17.5) / 47 (82.5)
Portal vein invasion (absent/present), n (%)	35 (61.4) / 22 (38.6)
Extrahepatic spread (absent/present), n (%)	43 (75.4) / 14 (24.6)
Median serum AFP level (ng/ml, range)	491 (21–315700)
Sorafenib starting dosage (800/400 mg), n (%)	44 (77.2) / 13 (22.8)
Median observation period (days, range)	222 (47–810)

HCC, hepatocellular carcinoma; HBV, hepatitis B virus; HCV, hepatitis C virus; NBNC, non-HBV and non-HCV; ECOG, Eastern Cooperative Oncology Group; PS, performance status; AFP, alpha-fetoprotein.

### Sorafenib Treatment

The median duration of sorafenib treatment of the 57 HCC patients was 172 days (range, 10–660 days). Median sorafenib dosage per day within 2 weeks was 741 mg. Sorafenib was permanently discontinued in 51 patients. The most common cause of discontinuation was disease progression (n = 37, 64.9%), adverse events (n = 19, 33.3%), and patient preference (n = 1).

### Pre-Treatment Factors Associated with OS

The median observation period of the 57 HCC patients was 222 days (range, 47–810 days). According to univariate analysis, pre-treatment factors significantly associated with OS were the presence of portal vein invasion (hazard ratio [HR] = 2.439; 95% confidence interval [CI], 1.321–4.505; p = 0.0044) and serum AFP level (HR = 2.123; 95%CI, 1.109–4.065; p = 0.0231) (Table 2). Sorafenib starting dosage (800 mg vs. 400 mg) was not a significant factor associated with OS. According to multivariate analysis, the presence of portal vein invasion was the only significant and independent predictor of mortality (HR = 2.041; 95% CI, 1.067–3.891; p = 0.0310) (Table 2).

**Table 2:** Univariate and multivariate survival analyses according to pre-treatment factors.

	Univariate analysis		Multivariate analysis	
Factors	HR (95%CI)	*p* value	HR (95%CI)	*p* value
Age (<71 vs. ≥71 years)	1.419 (0.781–2.578)	0.2513		
Gender (female vs. male)	0.843 (0.404–1.759)	0.6484		
Etiology (HCV vs. Others)	1.454 (0.799–2.646)	0.2203		
ECOG PS (0 vs. 1)	0.614 (0.315–1.196)	0.1516		
Therapy for recurrence vs. initial therapy	0.954 (0.400–2.272)	0.9146		
Child-Pugh score (5 vs. 6)	0.940 (0.514–1.716)	0.8392		
Tumor size (<30 mm vs. ≥30 mm)	1.072 (0.586–1.962)	0.8215		
Number of tumors (≥4 vs. <4)	1.776 (0.785–4.020)	0.1680		
Portal vein invasion (present vs absent)	2.439 (1.321–4.505)	0.0044	2.041 (1.067–3.891)	0.0310
Extrahepatic metastasis (present vs. absent)	1.686 (0.863–3.295)	0.1264		
Serum AFP level (≥200 ng/ml vs. <200 ng/ml)	2.123 (1.109–4.065)	0.0231	1.675 (0.850–3.367)	0.1345
Sorafenib starting dosage (800 mg vs. 400 mg)	1.025 (0.526–1.997)	0.9429		

HR, hazard ratio; CI, confidence interval; HCV, hepatitis C virus; ECOG, Eastern Cooperative Oncology Group; PS, performance status; AFP, alpha-fetoprotein.

### Anti-Tumor Response and Cumulative OS According to Modified RECIST at 6 Weeks after Sorafenib Therapy

**Fig 1:**
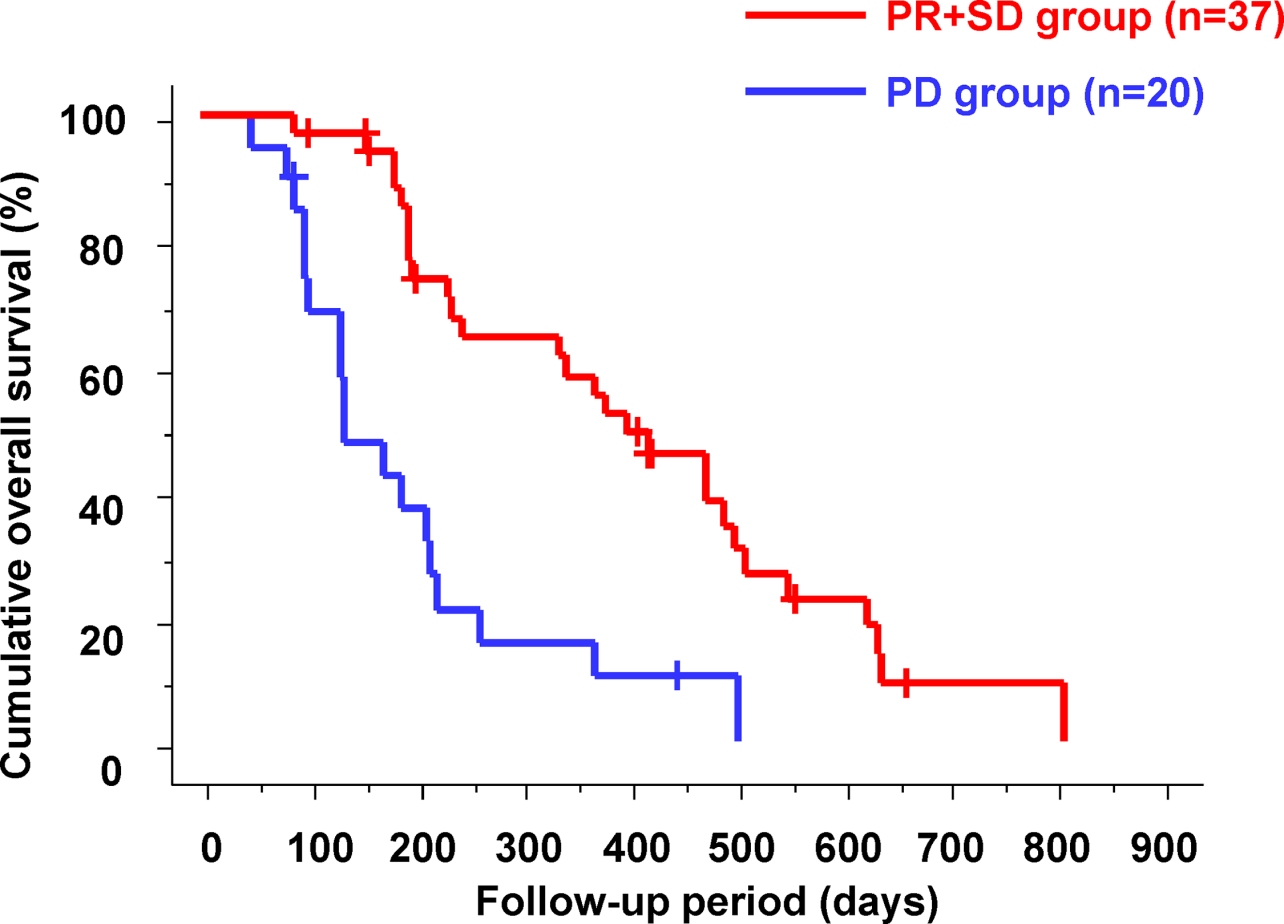
Cumulative overall survival according to modified RECIST. The median overall survival was significantly shorter in the progressive disease (PD) group than in the partial response (PR) + stable disease (SD) group (134 days vs. 378 days; p<0.0001).

Patients were classified into the partial response (PR), stable disease (SD), and progressive disease (PD) groups according to their response at 6 weeks after sorafenib administration according to modified RECIST. There were 11 patients (19.3%) in the PR group, 26 patients (45.6%) in the SD group, and 20 patients (35.1%) in the PD group. The response rate was 19.3%, and the disease control rate was 64.9%. The median OS was 423 days in the PR group, 369 days in the SD group, and 134 days in the PD group (p<0.0001). There were no significant differences in the cumulative OS of patients between the PR and SD groups (p = 0.3021). Cumulative OS in the PR+SD and PD groups is shown in Fig 1. The median OS was significantly shorter in the PD group than in the PR+SD group (134 days vs. 378 days; p<0.0001).

### Relationship between Changes in the Intra-Tumor Blood Flow at 2 Weeks after the Start of Sorafenib Therapy and Outcomes

**Fig 2:**
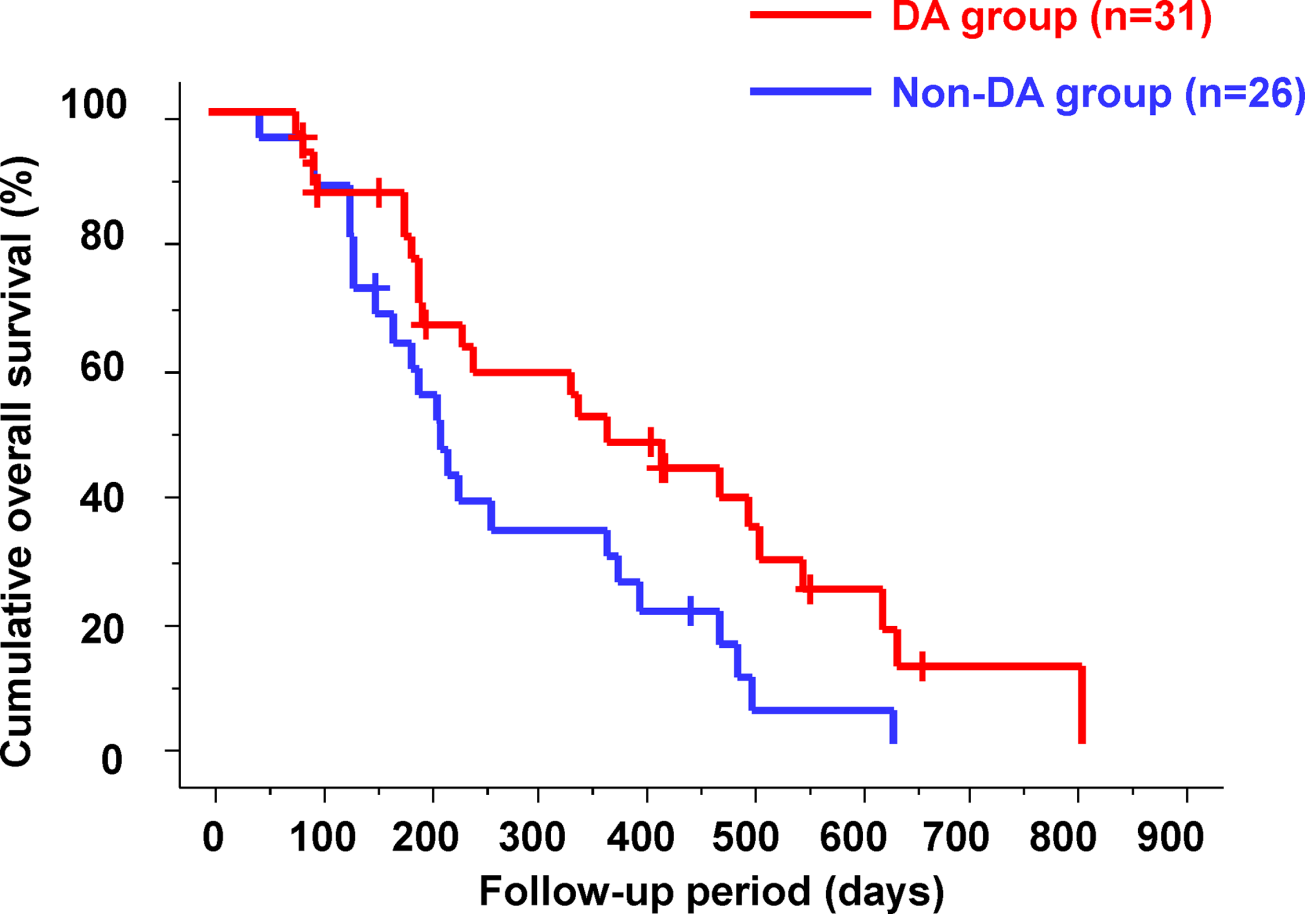
Cumulative overall survival according to the changes in the intra-tumor blood flow. The median overall survival was significantly shorter in the intra-tumor blood flow non-disappearance (non-DA) group than in the disappearance (DA) group (212 days vs. 341 days; p = 0.0204).

Patients were classified into the following two groups: (1) patients whose intra-tumor blood flow disappeared (the DA group) and (2) patients whose intra-tumor blood flow did not disappear (the non-DA group) at 2 weeks after the start of sorafenib therapy. There were 31 patients (54.4%) in the DA group and 26 patients (45.6%) in the non-DA group. Cumulative OS in the two groups according to the changes in the intra-tumor blood flow is shown in Fig 2. The median OS was significantly shorter in the non-DA group than in the DA group (212 days vs. 341 days; p = 0.0204).

### Relationship between the Changes in AFP Level at 2 Weeks after the Start of Sorafenib Therapy and Outcomes

**Fig 3:**
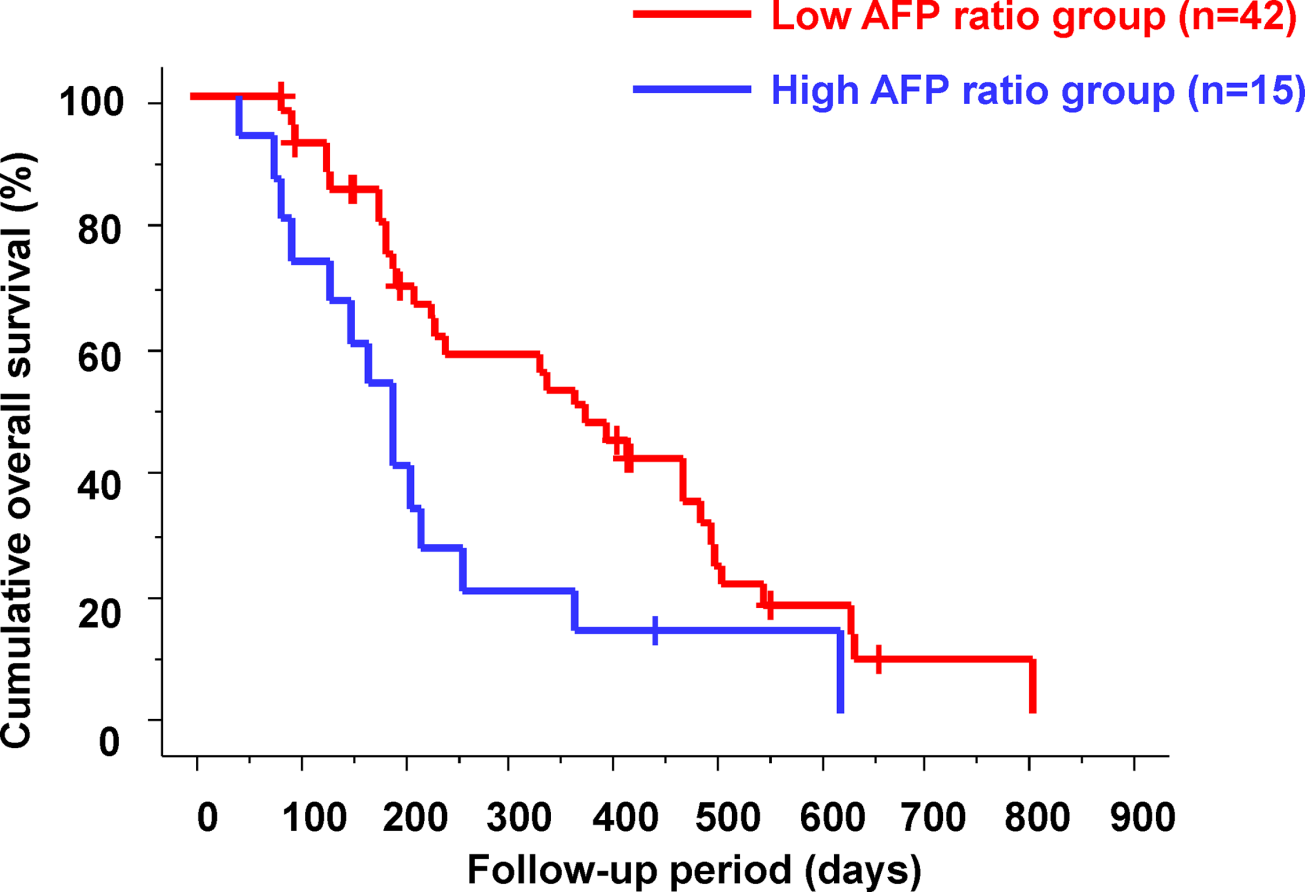
Cumulative overall survival according to changes in alpha-fetoprotein level. The median overall survival was significantly shorter in the high alpha-fetoprotein (AFP) ratio group than in the low AFP ratio group (170 days vs. 340 days; p = 0.0098).

The patients were subdivided into two groups: (1) patients with an AFP ratio of ≤1.2 (the low AFP ratio group) and (2) patients with an AFP ratio of >1.2 (the high AFP ratio group) at 2 weeks after the start of sorafenib therapy. There were 42 patients (73.7%) in the low AFP ratio group and 15 patients (26.3%) in the high AFP ratio group. Cumulative OS in the two groups according to changes in AFP level is shown in Fig 3. The median OS was significantly shorter in the high AFP ratio group than in the low AFP ratio group (170 days vs. 340 days; p = 0.0098).

### Relationship between Changes in Remnant Liver Function at 2 Weeks after the Start of Sorafenib Therapy and Outcomes

There were 25 patients (43.9%) with 1 or more increments in the CP score and 32 patients (56.1%) without increments in the CP score at 2 weeks after sorafenib administration. There were no significant differences in the cumulative OS of patients when comparing these two groups (p = 0.3968).

**Fig 4:**
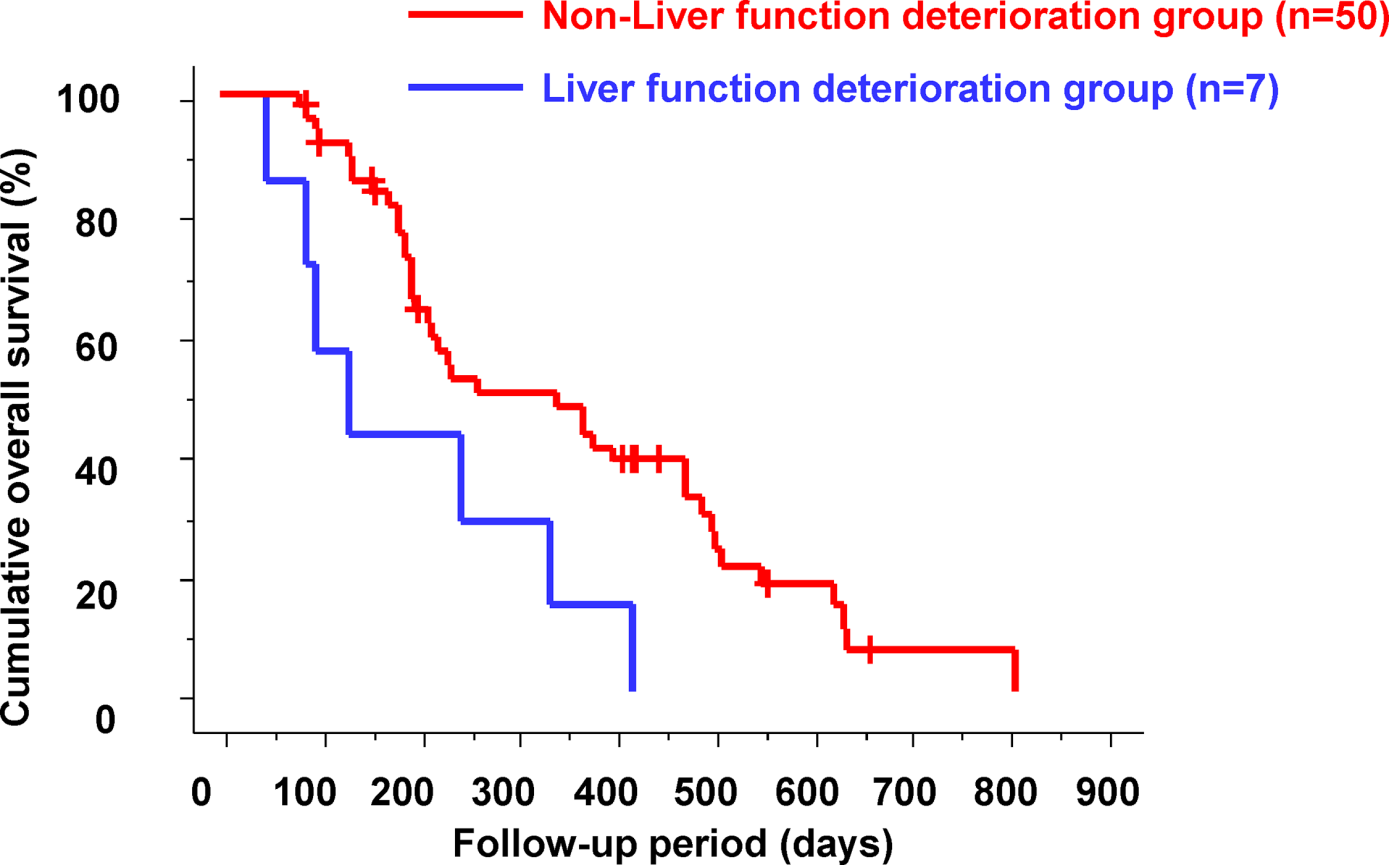
Cumulative overall survival according to changes in remnant liver function. The median overall survival was significantly shorter in the liver function deterioration group than in the non-liver function deterioration group (96 days vs. 263 days; p = 0.0111).

The patients were subdivided into two groups: (1) patients with 2 or more increments in the CP score at 2 weeks after sorafenib administration (liver function deterioration group) and (2) patients with less than 2 increments in the CP score (non-liver function deterioration group). There were seven patients (three patients with pretreatment CP score of 5; four patients with pretreatment CP score of 6) (12.3%) in the liver function deterioration group and 50 patients (87.7%) in the non-liver function deterioration group. Cumulative OS in the two groups according to changes in remnant liver function is shown in Fig 4. The median OS was significantly shorter in the liver function deterioration group than in the non-liver function deterioration group (96 days vs. 263 days; p = 0.0111).

### On-Treatment Factors at 2 Weeks after the Start of Sorafenib Therapy Associated with OS

According to univariate analysis, the on-treatment factors at 2 weeks after the start of sorafenib therapy significantly associated with worse OS were the absence of disappearance of arterial tumor enhancement on CE-CT (HR = 2.010; 95%CI, 1.099–3.673; p = 0.0233), AFP ratio of >1.2 (HR = 2.288; 95%CI, 1.196–4.386; p = 0.0123), and CP score elevation of ≥2 points (HR = 2.799; 95%CI, 1.216–6.440; p = 0.0155). Median sorafenib dosage within 2 weeks (<741 mg vs. ≥741 mg) was not a significant factor associated with OS (Table 3). According to multivariate analysis, the absence of disappearance of arterial tumor enhancement on CE-CT (HR = 2.121; 95%CI 1.079–4.170; p = 0.0292), AFP ratio of >1.2 (HR = 2.179; 95%CI 1.076–4.425; p = 0.0305), and CP score elevation of ≥2 points (HR = 4.729; 95%CI, 1.893–11.815; p = 0.0009) were significant and independent predictors of elevated mortality (Table 3).

**Table 3:** Univariate and multivariate survival analyses according to on-treatment factors at 2 weeks after the start of sorafenib therapy.

	Univariate analysis		Multivariate analysis	
Factors	HR (95%CI)	*p* value	HR (95%CI)	*p* value
Disappearance of arterial tumor enhancement on CE-CT (absent vs. present)	2.010 (1.099–3.673)	0.0233	2.121 (1.079–4.170)	0.0292
AFP ratio (>1.2 vs. ≤1.2)	2.288 (1.196–4.386)	0.0123	2.179 (1.076–4.425)	0.0305
Two or more increments in the CP score (present vs. absent)	2.799 (1.216–6.440)	0.0155	4.729 (1.893–11.815)	0.0009
Median sorafenib dosage within 2 weeks (<741 mg vs. ≥741 mg)	1.234 (0.683–2.230)	0.4869		

HR, hazard ratio; CI, confidence interval; CE-CT, contrast-enhanced computed tomography; AFP, alpha-fetoprotein; CP, Child-Pugh.

### Relationship between the Combination of Early Clinical Responses at 2 Weeks after the Start of Sorafenib Therapy and Outcomes

**Fig 5:**
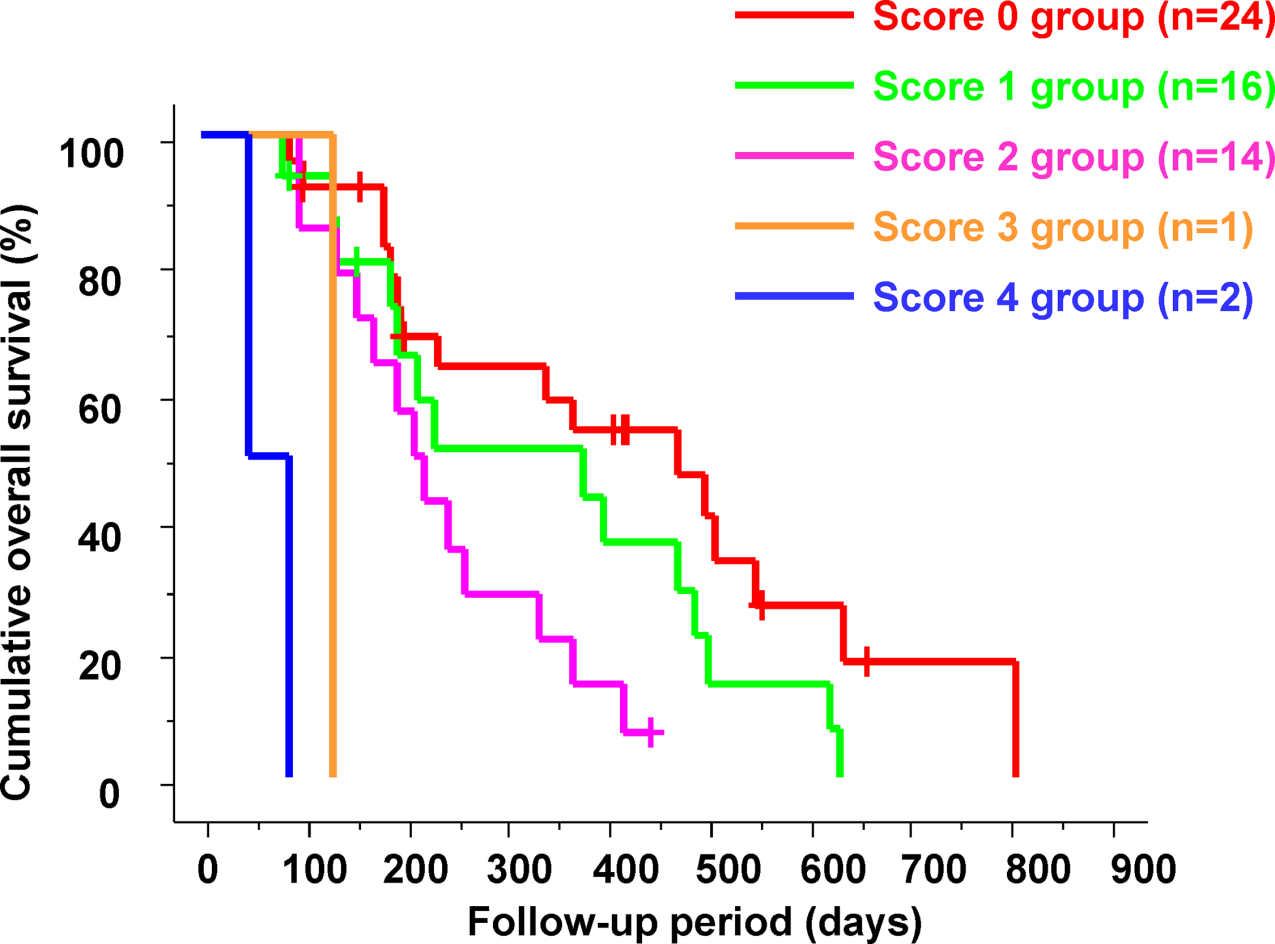
Cumulative overall survival in the poor prognostic score 0, 1, 2, 3, and 4 groups. There were significant differences in the cumulative overall survival of patients among the five groups (p<0.0001).

Patients received a score based on how many of the three variables identified in multivariate analysis as factors of poor prognosis (absence of disappearance of arterial tumor enhancement on CE-CT, AFP ratio of >1.2, CP score elevation of ≥2 points) that they had. This score was referred to as "poor prognostic score at 2 weeks" and was attributed to each variable according to its relative contribution in the Cox proportional hazard model. A sum poor prognostic score of the change in intra-tumor blood flow (DA = 0, non-DA = 1), the AFP level (low AFP ratio = 0, high AFP ratio = 1) and remnant liver function (non-liver function deterioration = 0, liver function deterioration = 2) at 2 weeks after the start of sorafenib therapy was calculated. The patients were divided into five groups based on the poor prognostic score. There were 24 patients (42.1%) in the poor prognostic score 0 group, 16 patients (28.7%) in the poor prognostic score 1 group, 14 patients (24.6%) in the poor prognostic score 2 group, one patient (1.8%) in the poor prognostic score 3 group, and two patients (3.5%) in the poor prognostic score 4 group after 2 weeks of sorafenib therapy. Cumulative OS in the five groups according to these poor prognostic scores is shown in Fig 5. There were significant differences in the cumulative OS of patients among the five groups (p<0.0001).

**Fig 6:**
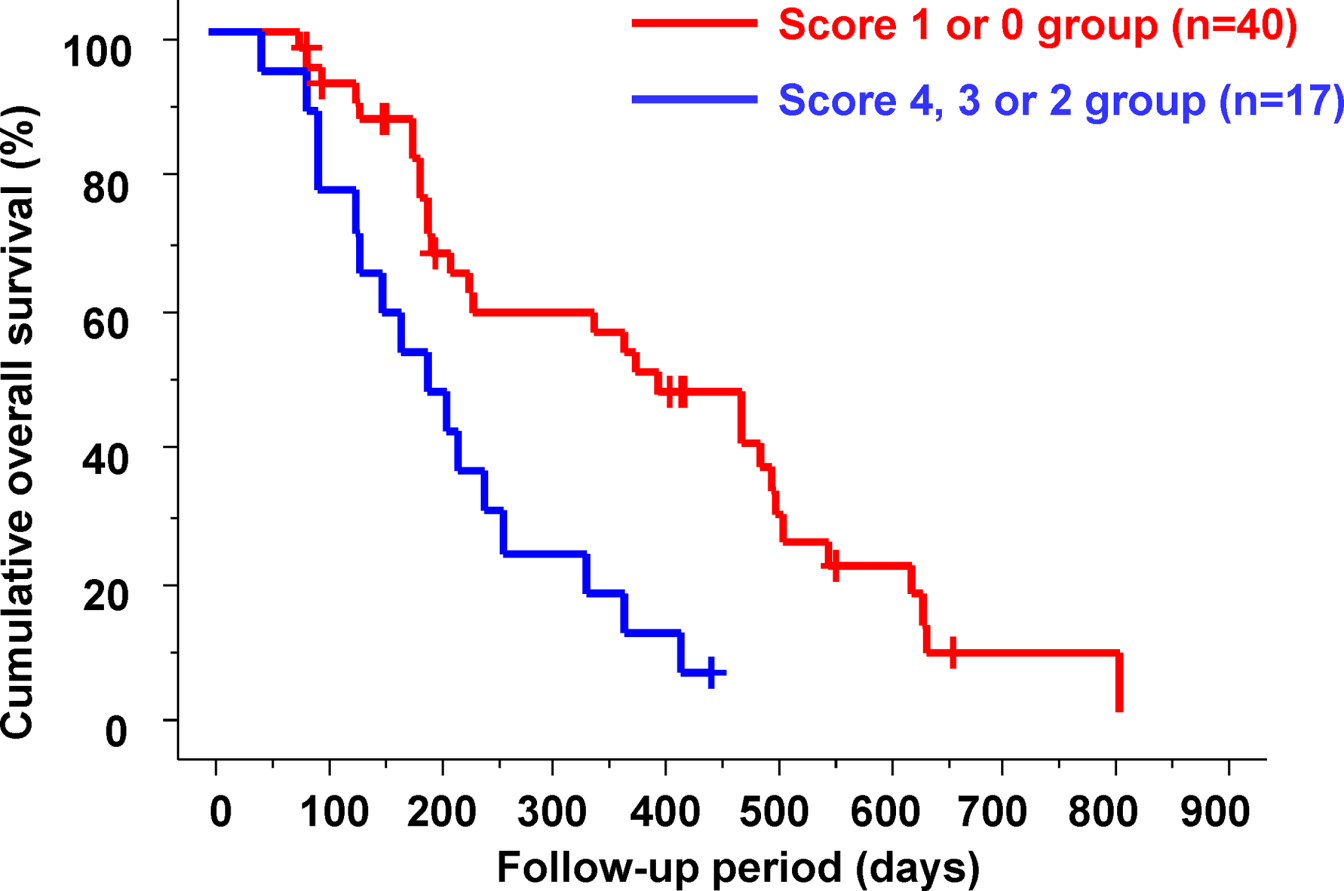
Cumulative overall survival in the poor prognostic score 4, 3 or 2 and 1 or 0 groups. In the poor prognostic score 4, 3 or 2 groups, the median overall survival was significantly shorter than that in the poor prognostic score 1 or 0 groups (194 vs. 378 days; p = 0.0010).

There were 40 patients (70.2%) in the poor prognostic score 1 or 0 group and 17 patients (29.8%) in the poor prognostic score 4, 3 or 2 group after 2 weeks of sorafenib therapy. Cumulative OS in the poor prognostic score 4, 3 or 2 and 1 or 0 groups is shown in Fig 6. In the poor prognostic score 4, 3 or 2 groups, the median OS was significantly shorter than that in the poor prognostic score 1 or 0 groups (194 vs. 378 days; p = 0.0010).

### Relationship between the Poor Prognostic Score at 2 Weeks and the Anti-Tumor Effect at 6 Weeks

The relationship between the each prognostic score at 2 weeks and the anti-tumor effect at 6 weeks by modified RECIST is shown in Table 4. A poor prognostic score of 0 (n = 24) was recorded in 22 PR+SD patients and in two PD patients. A poor prognostic score of 1 (n = 16) was recorded in 10 PR+SD patients and in six PD patients. A poor prognostic score of 2 (n = 14) was recorded in five PR+SD patients and nine PD patients. A poor prognostic score of 3 (n = 1) was recorded in one PD patient. A poor prognostic score of 4 (n = 2) was recorded in zero PR+SD patients and in two PD patients. The relationship between the two stratified prognostic score groups at 2 weeks and the anti-tumor effect at 6 weeks by modified RECIST is shown in Table 5. A poor prognostic score of 1 or 0 (n = 40) was recorded in 32 PR+SD patients and in eight PD patients. A poor prognostic score of 4, 3 or 2 (n = 17) was recorded in five PR+SD patients and 12 PD patients. The PD rate in patients with a poor prognostic score of 4, 3 or 2 was significantly higher than that in patients with a poor prognostic score of 1 or 0 (70.6% vs. 20.0%, p = 0.0003).

**Table 4:** Relationship between the each poor prognostic score at 2 weeks and the anti-tumor response by modified RECIST at 6 weeks.

	Poor prognostic score at 2 weeks, n (%)				
	Score 0 n = 24	Score 1 n = 16	Score 2 n = 14	Score 3 n = 1	Score 4 n = 2
**PR+SD by modified RECIST at 6 weeks**	22 (91.7)	10 (62.5)	5 (35.7)	0 (0.0)	0 (0.0)
**PD by modified RECIST at 6 weeks**	2 (8.3)	6 (37.5)	9 (64.3)	1 (100.0)	2 (100.0)

**Table 5:** Relationship between the two stratified poor prognostic score groups at 2 weeks and the anti-tumor response by modified RECIST at 6 weeks.

	Poor prognostic score at 2 weeks, n (%)	
	Score 1 or 0 n = 40	Score 4, 3 or 2 n = 17
**PR+SD by modified RECIST at 6 weeks**	32 (80.0)	5 (29.4)
**PD by modified RECIST at 6 weeks**	8 (20.0)	12 (70.6)

PR; partial response, SD; stable disease, RECIST; Response Evaluation Criteria in Solid Tumors, PD; progressive disease.

## Discussion

In the present study, we investigated the relationship between the early clinical response in intra-tumor blood flow on CE-CT, AFP levels and remnant liver function after 2 weeks of sorafenib treatment and the outcomes and anti-tumor response in patients with advanced HCC. We found that the absence of disappearance of arterial tumor enhancement on CE-CT, AFP ratio of >1.2, and two or more increments in the CP score after 2 weeks of sorafenib therapy were significant and independent predictors of worse survival and that these three worse OS factors (the absence of disappearance of arterial tumor enhancement on CE-CT, AFP ratio of >1.2, and two or more increments in the CP score) were also associated with a significantly higher PD rate according to modified RECIST at 6 weeks after sorafenib therapy.

Reductions in intra-tumor blood flow are often observed after sorafenib therapy without measurable change in tumor size [3,15,20]. Some reports have documented that OS is better reflected by the modified RECIST based on tumor viability measurement than by the conventional RECIST based on tumor burden measurement for patients receiving sorafenib therapy [29,30]. Therefore, it is important to evaluate whether or not a disappearance of arterial tumor enhancement has occurred in order to evaluate the efficacy of sorafenib therapy.

In general, regarding the timing to evaluate treatment response, the formal recommendation of the panel of experts in the HCC-Design Clinical Trials was to conduct imaging surveillance every 6 to 8 weeks using CT or magnetic resonance imaging [7]. In the present study, in order to assess the early radiological image therapeutic effects, we focused on whether or not a disappearance of arterial tumor enhancement in at least one lesion of target lesions after 2 weeks of sorafenib therapy occurred. We found that 26 patients (45.6%) did not have disappearance of arterial tumor enhancement on CE-CT at 2 weeks after sorafenib administration, and this observation correlated with worse OS. Further, by univariate and multivariate analyses, the absence of disappearance of arterial tumor enhancement on CE-CT was significantly associated with worse OS. Therefore, the present results indicate that it may be beneficial to evaluate for the disappearance of arterial tumor enhancement on CE-CT time point at 2 weeks after sorafenib administration. To our knowledge, this is the first study to evaluate the radiological response on CE-CT imaging after 2 weeks of sorafenib therapy and to investigate its relationship with outcomes.

Some reports suggest that early AFP response after sorafenib therapy is a useful surrogate marker for anti-tumor response and outcomes [18–21,24,27,28]. Personeni et al. reported that a >20% decline of AFP after 8 weeks of sorafenib treatment was significantly associated with better outcomes [19]. Takeda et al. reported that a >20% decrease in the AFP levels at 1 month after the initiation of sorafenib therapy was an independent predictor of a favorable radiological response (complete or partial response according to modified RECIST) [21]. Nakazawa et al. reported that a >20% increase in the AFP levels within 4 weeks after the initiation of sorafenib was significantly related to PD and was a significant independent predictor of both poor OS and progression-free survival [27]. Consistent with these reports, the present study showed that early AFP response after 2 weeks of sorafenib therapy was significantly associated with OS.

With respect to the relationship between the prognosis and the remnant liver function in sorafenib therapy, several reports have documented that remnant liver function is a significant prognostic factor [20,31–33]. Okuyama et al. reported that presence of ascites and elevated serum total bilirubin level at the time of discontinuation of sorafenib therapy were independent unfavorable prognostic factors in patients with advanced HCC who were refractory or intolerant to sorafenib [33]. To our knowledge, there are no reports regarding the relationship between prognosis and the changes in remnant liver function in the very early period following administration of sorafenib. In the present study, the deterioration of remnant liver function (two or more increments of CP score) at 2 weeks after sorafenib therapy was the most significant and independent predictor of mortality. Therefore, according to our present results, early and careful monitoring via medical examination and hepatic function tests is necessary to avoid deterioration of remnant liver function in the very early period after initiating sorafenib therapy.

Taken together, in the present study, we found that the absence of disappearance of arterial tumor enhancement on CE-CT, AFP ratio of >1.2, and two or more increments in the CP score after 2 weeks of sorafenib therapy were significant and independent predictors of worse survival and that these three worse prognostic factors were also associated with a significantly higher PD rate according to modified RECIST at 6 weeks after sorafenib therapy. Therefore, our results suggest that sorafenib therapy may be considered ineffective if patients have these three adverse prognostic factors at 2 weeks after sorafenib administration. These adverse prognostic factors may be useful to identify patients who are unsuitable for continuation of sorafenib therapy.

It is important to identify whether sorafenib therapy is beneficial or not as early as possible, as appropriate and early evaluation of sorafenib therapy can identify patients in whom such therapy should be terminated. This would avoid needless adverse events and costs, and allow second-line therapy when sorafenib therapy is not effective. At present, there is no established second-line treatment for advanced HCC, and we could not confirm whether continuing sorafenib administration would prolong the survival of patients with elevated AFP level and with no radiological response at 2 weeks after sorafenib therapy. Accordingly, we cannot conclude that sorafenib therapy should be stopped in the case of elevation of AFP level and/or no radiological response after 2 weeks of sorafenib therapy. However, when an effective second-line treatment becomes available, the present results may be used to guide a switch to second-line therapy.

Sorafenib is often recommended as a treatment option for transarterial chemoembolization (TACE)-refractory patients [3,4]. However, in clinical practice, doctors are sometimes unsure whether sorafenib treatment is indicated in some TACE-refractory patients because of the modest treatment response, the high cost, and the high incidence of drug-related adverse events. Identification of patients who are expected to benefit from sorafenib therapy in the very early period following administration of sorafenib may be helpful when considering a switch from TACE to sorafenib therapy in TACE-refractory patients. In addition, evaluation of early clinical response may be useful when assessing the efficacy of other molecular targeted agents that are currently under development.

There were several limitations in the present study. First, this was a multicenter study, so CE-CT imaging results could have varied between study sites. All study sites performed the CE-CT imaging in four phases, but each site used different CT devices and contrast agents, so it is possible that the results were not entirely consistent. Second, this study only investigated changes in intra-tumor blood flow on CE-CT in patients who had hypervascular tumors at baseline, so the study outcomes are not applicable to patients with only hypovascular tumors or those with only distant metastases at the start of sorafenib therapy. Third, we investigated only whether or not there was disappearance of arterial tumor enhancement on CE-CT at 2 weeks post-dose in order to evaluate the changes in intra-tumor blood flow, because only the disappearance of arterial tumor enhancement is taken into consideration in the modified RECIST. However, in clinical practice, a disappearance or a decrease of arterial tumor enhancement is often observed, and one report documented that patients with either disappearance or a decrease in tumor enhancement showed better OS than patients without disappearance or a decrease in tumor enhancement [15]. Therefore, it may be preferable to investigate changes in intra-tumor blood flow as a continuous variable by using changes in quantitative enhancement on CE-CT rather than evaluating this parameter as a dichotomous variable. Fourth, this study only investigated changes in AFP levels in patients with abnormal AFP levels (i.e., serum AFP ≥20 ng/ml) at the start of sorafenib therapy, so the study outcomes are not applicable to patients whose baseline AFP levels are within the normal range (i.e., serum AFP <20 ng/ml). Fifth, in the several reports that investigated the relationship between AFP response and favorable outcomes to sorafenib therapy, 0.8 is often used as a cut-off of the AFP ratio [18,19,21]. On the other hand, there are only a few reports that investigated the relationship between the AFP response and unfavorable outcomes. We adopted 1.2 (as was used in two reports [27,28]) as the cut-off of AFP ratio at 2 weeks post-dose. In the present study, an AFP ratio of 1.2 at 2 weeks post-dose was an independent and significantly poor prognostic factor according to both univariate and multivariable analysis. In future studies, it is necessary to confirm whether 1.2 is the optimal AFP cut-off for this purpose. Finally, the present study had a small sample. Therefore, confirmation of our findings requires further studies with larger numbers of patients. Furthermore, study of an independent cohort is necessary to validate our results.

In conclusion, our results suggest that changes in intra-tumor blood flow, AFP levels and remnant liver function after 2 weeks of sorafenib therapy may be useful for predicting outcomes and anti-tumor response in patients with advanced HCC. Identification of patients who are expected to benefit from sorafenib therapy in the very early period following administration of sorafenib can provide valuable information that may influence subsequent treatment strategy decisions and help avoid unnecessary adverse events and costs in patients who would not benefit from ongoing sorafenib therapy.

## References

[1] Llovet JM, Ricci S, Mazzaferro V, Hilgard P, Gane E, Blanc JF, et al Sorafenib in advanced hepatocellular carcinoma. N Engl J Med. 2008; 359: 378–390. 10.1056/NEJMoa0708857 18650514

[2] Cheng AL, Kang YK, Chen Z, Tsao CJ, Qin S, Kim JS, et al Efficacy and safety of sorafenib in patients in the Asia-Pacific region with advanced hepatocellular carcinoma: a phase III randomised, double-blind, placebo-controlled trial. Lancet Oncol. 2009; 10: 25–34. 10.1016/S1470-2045(08)70285-7 19095497

[3] Kaneko S, Furuse J, Kudo M, Ikeda K, Honda M, Nakamoto Y, et al Guideline on the use of new anticancer drugs for the treatment of Hepatocellular Carcinoma 2010 update. Hepatol Res. 2012; 42: 523–542. 10.1111/j.1872-034X.2012.00981.x 22568457

[4] Kudo M, Izumi N, Kokudo N, Matsui O, Sakamoto M, Nakashima O, et al Management of hepatocellular carcinoma in Japan: Consensus-Based Clinical Practice Guidelines proposed by the Japan Society of Hepatology (JSH) 2010 updated version. Dig Dis. 2011; 29: 339–364. 10.1159/000327577 21829027

[5] Bruix J, Gores GJ, Mazzaferro V. Hepatocellular carcinoma: clinical frontiers and perspectives. Gut. 2014; 63: 844–855. 10.1136/gutjnl-2013-306627 24531850 PMC4337888

[6] Colombo M, Sangiovanni A. Treatment of hepatocellular carcinoma: beyond international guidelines. Liver Int. 2015; 35: 129–138. 10.1111/liv.12713 25529098

[7] Llovet JM, Di Bisceglie AM, Bruix J, Kramer BS, Lencioni R, Zhu AX, et al Design and endpoints of clinical trials in hepatocellular carcinoma. J Natl Cancer Inst. 2008; 100: 698–711. 10.1093/jnci/djn134 18477802

[8] Reig M, Torres F, Rodriguez-Lope C, Forner A, LLarch N, Rimola J et al Early dermatologic adverse events predict better outcome in HCC patients treated with sorafenib. J Hepatol. 2014; 61: 318–324. 10.1016/j.jhep.2014.03.030 24703956

[9] Cammà C, Cabibbo G, Petta S, Enea M, Iavarone M, Grieco A, et al Cost-effectiveness of sorafenib treatment in field practice for patients with hepatocellular carcinoma. Hepatology. 2013; 57: 1046–1054. 10.1002/hep.26221 23299720

[10] Bruix J, Raoul JL, Sherman M, Mazzaferro V, Bolondi L, Craxi A, et al Efficacy and safety of sorafenib in patients with advanced hepatocellular carcinoma: subanalyses of a phase III trial. J Hepatol. 2012; 57: 821–829. 10.1016/j.jhep.2012.06.014 22727733 PMC12261288

[11] Cheng AL, Guan Z, Chen Z, Tsao CJ, Qin S, Kim JS, et al Efficacy and safety of sorafenib in patients with advanced hepatocellular carcinoma according to baseline status: subset analyses of the phase III Sorafenib Asia-Pacific trial. Eur J Cancer. 2012; 48: 1452–1465. 10.1016/j.ejca.2011.12.006 22240282

[12] Llovet JM, Peña CE, Lathia CD, Shan M, Meinhardt G, Bruix J. Plasma biomarkers as predictors of outcome in patients with advanced hepatocellular carcinoma. Clin Cancer Res. 2012; 18: 2290–2300. 10.1158/1078-0432.CCR-11-2175 22374331 PMC12268944

[13] Shao YY, Hsu CH, Cheng AL. Predictive biomarkers of antiangiogenic therapy for advanced hepatocellular carcinoma: where are we? Liver Cancer. 2013; 2: 93–107. 10.1159/000343845 24159601 PMC3740718

[14] Miyahara K, Nouso K, Tomoda T, Kobayashi S, Hagihara H, Kuwaki K, et al Predicting the treatment effect of sorafenib using serum angiogenesis markers in patients with hepatocellular carcinoma. J Gastroenterol Hepatol. 2011; 26: 1604–1611. 10.1111/j.1440-1746.2011.06887.x 22011296

[15] Arizumi T, Ueshima K, Chishina H, Kono M, Takita M, Kitai S, et al Decreased blood flow after sorafenib administration is an imaging biomarker to predict overall survival in patients with advanced hepatocellular carcinoma. Dig Dis. 2014; 32: 733–739. 10.1159/000368013 25376291

[16] Sugimoto K, Moriyasu F, Saito K, Rognin N, Kamiyama N, Furuichi Y, et al Hepatocellular carcinoma treated with sorafenib: early detection of treatment response and major adverse events by contrast-enhanced US. Liver Int. 2013; 33: 605–615. 10.1111/liv.12098 23305331

[17] Zocco MA, Garcovich M, Lupascu A, Di Stasio E, Roccarina D, Annicchiarico BE, et al Early prediction of response to sorafenib in patients with advanced hepatocellular carcinoma: the role of dynamic contrast enhanced ultrasound. J Hepatol. 2013; 59: 1014–1021. 10.1016/j.jhep.2013.06.011 23811306

[18] Shao YY, Lin ZZ, Hsu C, Shen YC, Hsu CH, Cheng AL. Early alpha-fetoprotein response predicts treatment efficacy of antiangiogenic systemic therapy in patients with advanced hepatocellular carcinoma. Cancer. 2010; 116: 4590–4596. 10.1002/cncr.25257 20572033

[19] Personeni N, Bozzarelli S, Pressiani T, Rimassa L, Tronconi MC, Sclafani F, et al Usefulness of alpha-fetoprotein response in patients treated with sorafenib for advanced hepatocellular carcinoma. J Hepatol. 2012; 57: 101–107. 10.1016/j.jhep.2012.02.016 22414760

[20] Kawaoka T, Aikata H, Murakami E, Nakahara T, Naeshiro N, Tanaka M, et al Evaluation of the mRECIST and α-fetoprotein ratio for stratification of the prognosis of advanced-hepatocellular-carcinoma patients treated with sorafenib. Oncology. 2012; 83: 192–200. 10.1159/000341347 22890083

[21] Takeda H, Nishikawa H, Osaki Y, Tsuchiya K, Joko K, Ogawa C, et al Clinical features associated with radiological response to sorafenib in unresectable hepatocellular carcinoma: a large multicenter study in Japan. Liver Int. 2015; 35: 1581–1589. 10.1111/liv.12591 24836552

[22] Tsuchiya K, Asahina Y, Matsuda S, Muraoka M, Nakata T, Suzuki Y, et al Changes in plasma vascular endothelial growth factor at 8 weeks after sorafenib administration as predictors of survival for advanced hepatocellular carcinoma. Cancer. 2014; 120: 229–237. 10.1002/cncr.28384 24122122 PMC4209122

[23] Kuzuya T, Ishigami M, Niinomi T, Imai N, Achiwa K, Arakawa T, et al The relationship between the fever and the disappearance or decrease of lesion contrast on contrast enhanced CT images after 2 weeks of sorafenib treatment for patients with advanced hepatocellular carcinoma. Kanzo. 2013; 54: 505–506.

[24] Kuzuya T, Asahina Y, Tsuchiya K, Tanaka K, Suzuki Y, Hoshioka T, et al Early decrease in α-fetoprotein, but not des-γ-carboxy prothrombin, predicts sorafenib efficacy in patients with advanced hepatocellular carcinoma. Oncology. 2011; 81: 251–258. 10.1159/000334454 22116493

[25] Bruix J, Sherman M. American Association for the Study of Liver Diseases. Management of hepatocellular carcinoma: an update. Hepatology. 2011; 53: 1020–1022. 10.1002/hep.24199 21374666 PMC3084991

[26] Lencioni R, Llovet JM. Modified RECIST (mRECIST) Assessment for Hepatocellular Carcinoma. Semin Liver Dis. 2010; 30: 52–60. 10.1055/s-0030-1247132 20175033 PMC12268942

[27] Nakazawa T, Hidaka H, Takada J, Okuwaki Y, Tanaka Y, Watanabe M, et al Early increase in α-fetoprotein for predicting unfavorable clinical outcomes in patients with advanced hepatocellular carcinoma treated with sorafenib. Eur J Gastroenterol Hepatol. 2013; 25: 683–689. 10.1097/MEG.0b013e32835d913b 23395995

[28] Ogasawara S, Chiba T, Ooka Y, Kanogawa N, Saito T, Motoyama T, et al Sorafenib treatment in Child-Pugh A and B patients with advanced hepatocellular carcinoma: safety, efficacy and prognostic factors. Invest New Drugs. 2015; 33: 729–739. 10.1007/s10637-015-0237-3 25861764

[29] Edeline J, Boucher E, Rolland Y, Vauléon E, Pracht M, Perrin C, et al Comparison of tumor response by Response Evaluation Criteria in Solid Tumors (RECIST) and modified RECIST in patients treated with sorafenib for hepatocellular carcinoma. Cancer. 2012; 118: 147–156. 10.1002/cncr.26255 21713764

[30] Arizumi T, Ueshima K, Takeda H, Osaki Y, Takita M, Inoue T, et al Comparison of systems for assessment of post-therapeutic response to sorafenib for hepatocellular carcinoma. J Gastroenterol. 2014; 49: 1578–1587. 10.1007/s00535-014-0936-0 24499826 PMC4258615

[31] Kim HY, Park JW, Joo J, Kim H, Woo SM, Lee WJ, et al Worse outcome of sorafenib therapy associated with ascites and Child-Pugh score in advanced hepatocellular carcinoma. J Gastroenterol Hepatol. 2013; 28: 1756–1761. 10.1111/jgh.12310 23800278

[32] Lee S, Kim BK, Kim SU, Park SY, Kim JK, Lee HW, et al Clinical outcomes and prognostic factors of patients with advanced hepatocellular carcinoma treated with sorafenib as first-line therapy: a Korean multicenter study. J Gastroenterol Hepatol. 2014; 29: 1463–1469. 10.1111/jgh.12542 25273508

[33] Okuyama H, Ikeda M, Kuwahara A, Takahashi H, Ohno I, Shimizu S, et al Prognostic Factors in Patients with Hepatocellular Carcinoma Refractory or Intolerant to Sorafenib. Oncology. 2014; 88: 241–246. 10.1159/000369351 25503567

